# Musculoskeletal diseases in Marfan syndrome: a nationwide registry study

**DOI:** 10.1186/s13023-022-02272-2

**Published:** 2022-03-05

**Authors:** Niels H. Andersen, Ellen-Margrethe Hauge, Thomas Baad-Hansen, Kristian A. Groth, Agnethe Berglund, Claus H. Gravholt, Kirstine Stochholm

**Affiliations:** 1 Department of Cardiology, Aalborg University Hospital, Hobrovej 18-22, 9000 Aalborg, Denmark; 2 Department of Rheumatology, Aarhus University Hospital, Palle Juul-Jensens Boulevard, 8200 Aarhus, Denmark; 3 Department of Clinical Medicine, Aarhus University, Incuba Skejby, Palle Juul-Jensens Boulevard, 8200 Aarhus, Denmark; 4 Department of Orthopedics, Aarhus University Hospital, Palle Juul-Jensens Boulevard, 8200 Aarhus, Denmark; 5 Department of Clinical Genetics, Aarhus University Hospital, Palle Juul-Jensens Boulevard, 8200 Aarhus, Denmark; 6 Department of Molecular Medicine, Aarhus University Hospital, Palle Juul-Jensens Boulevard, 8200 Aarhus, Denmark; 7 Department of Endocrinology and Internal Medicine, Aarhus University Hospital, Palle Juul-Jensens Boulevard, 8200 Aarhus, Denmark; 8 Center for Rare Diseases, Aarhus University Hospital, Palle Juul-Jensens Boulevard, 8200 Aarhus, Denmark

**Keywords:** Inherited disease, Musculoskeletal abnormalities, Spine, Pediatrics, Orthopedic surgery, Marfan syndrome

## Abstract

**Background:**

Marfan syndrome is associated with abnormalities in the musculoskeletal system including scoliosis, pectus deformities, protrusio acetabuli, and foot deformities. Over a life span, many patients with Marfan syndrome will need treatment; however, the musculoskeletal morbidity over a life span is not well described. The aim of the present study was to assess the overall burden of musculoskeletal disease in patients with Marfan syndrome.

**Materials and methods:**

A registry-based, nationwide epidemiological study of patients with a Ghent II verified Marfan syndrome diagnosis from 1977 to 2014. Each patient was matched on age, and sex with up to 100 controls from the background population.

**Results:**

We identified 407 patients with Marfan syndrome and 40,700 controls and compared their musculoskeletal diagnoses and surgical treatments using Cox proportional hazards ratio (HR). The risk of a registration of a musculoskeletal diagnosis in patients with Marfan syndrome was significantly increased compared to controls (HR: 1.94 (1.69–2.24). One out of six with Marfan syndrome was registered with scoliosis (HR: 36.7 (27.5–48.9). Scoliosis was more common in women with Marfan syndrome compared to men (HR: 4.30 (1.73–1.08)). One out of 11 were registered with a pectus deformity HR: 40.8 (28.1–59.3), and one out of six with a deformity of the foot. Primarily pes planus (HR: 26.0 (15.2–44.3). The proportion of patients with Marfan syndrome (94/407) that underwent musculoskeletal surgery was also significantly higher (HR: 1.76 (1.43–2.16)). The major areas of surgery were the spine, pectups correction, and surgery of the foot/ankle. Ten patients with Marfan syndrome had elective orthopedic surgery without being recognized and diagnosed with Marfan syndrome until later in life. None of these had scoliosis, pectus deformity or a foot deformity. Among patients with an aortic dissection, the age at dissection was 34.3 years in those with at least one major musculoskeletal abnormality. In patients without a major abnormality the age at dissection was 45.1 years (*p* < 0.01).

**Conclusions:**

The extent of musculoskeletal disease is quite significant in Marfan syndrome, and many will need corrective surgery during their life span. Surgeons should be aware of undiagnosed patients with Marfan syndrome when treating patients with a Marfan syndrome like-phenotype.

## Introduction

**Fig. 1:**
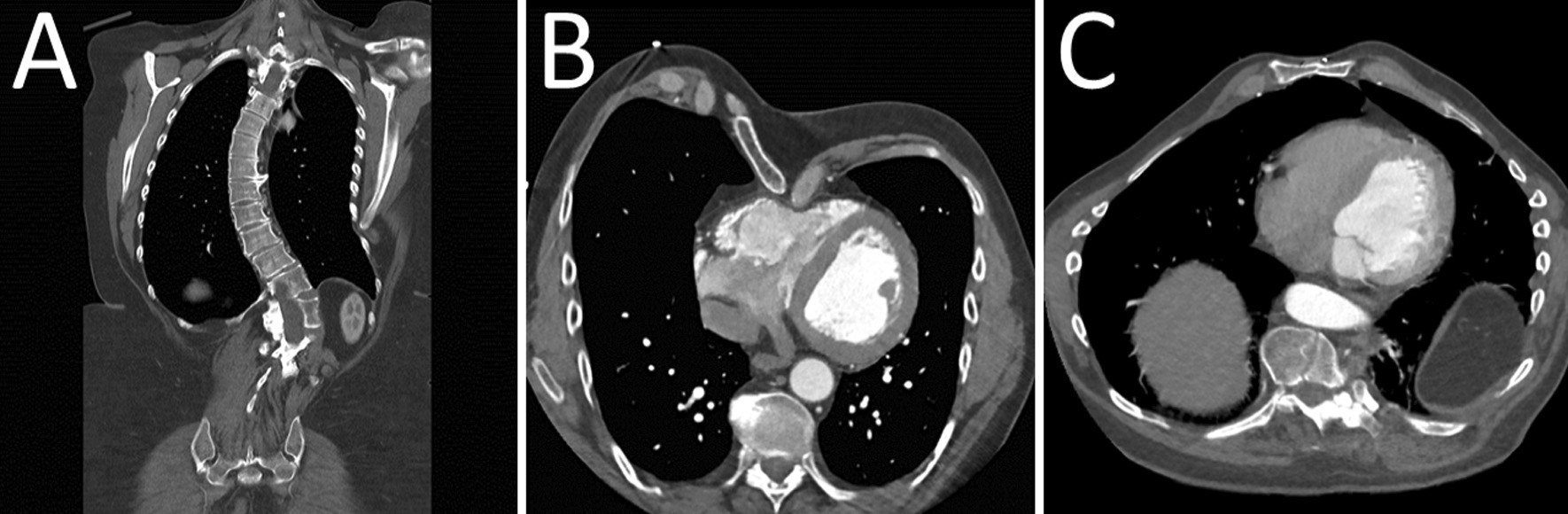
Skeletal abnormalities in two adults with Marfan syndrome. **A** 37-year-old women with new diagnosed Marfan syndrome. CT scan reveals a thoracic dextroscoliosis with osteophytes. **B** 56-year-old male with Marfan syndrome and severe pectus excavatum with compression of the right ventricle. Even though the patient was diagnosed as a child he was never offered corrective surgery of the chest. **C** 38-year-old male with pectus carinatum. He had never been interested in corrective surgery

Marfan syndrome (MFS; 154,700) is a genetic disorder with autosomal dominant heritage caused by pathological variants in the fibrillin-1 gene (*FBN1*; 134,797) [1]. The disease prevalence is 6.5/100,000 persons [2]. The diagnosis of a patient with MFS can be based on the presence of a pathogenic variant and disease in the ascending aorta or the eye lens [3]. Since MFS is also associated with numerous abnormalities in the musculoskeletal system including scoliosis, pectus deformities, protrusio acetabuli, and foot deformities. These impairments are also featured in the systemic criteria in the Ghent II nosology and play a significant part in the diagnosis of a patient with MFS [3, 4]. Beside the cardiac concerns, there is little doubt that the burdensome musculoskeletal morbidity is severely affecting persons with MFS already during childhood and adolescence [5, 6]. The causal mechanisms behind the musculoskeletal manifestations in MFS are quite complex, but the central mechanisms are reduced production of normal fibrillin-1 and interfered formation of fibrillin microfibrils which weaken connective tissue [7]. In combination with overgrowth of the long bones both in the extremities and the ribs, the patients primarily develop deformities of the spine, chest wall and feet [8] (Fig. 1). A recent study found a considerable number of musculoskeletal abnormalities in 1575 patients with MFS including scoliosis in 45%, pectus deformities in 46%, and pes planus in 39% [9]. Moreover, manifestations not found in the Ghent criteria, such as arachnodactyly and spondylolisthesis were also very common [9]. This huge burden of musculoskeletal disorders will affect most patients with MFS during their lifetime [10]. Many young patients will choose to have corrective surgery of the chest wall [11, 12] and others will need treatment of their scoliosis at some point in life [13]. The study aimed at assessing the overall burden of musculoskeletal disease in patients with MFS. By utilizing information from the Danish national health registries on all diagnoses and treatments related to the musculoskeletal system, we hereby describe the findings from all patients in Denmark with a Ghent-II verified diagnosis of MFS and compare them to an age and sex matched background population.

## Methods

All patients in Denmark with MFS were identified by use of Statistics Denmark.

Statistics Denmark is the central authority on most Danish national health care registries, including the Danish National Patient Registry [14]. The Danish registries are scientifically invaluable, as they hold information including dates and types of diagnoses on all registrations of in- and outpatients, dates and types of all surgeries, vital status, including date and cause of death and much more for all persons residing in Denmark. The health care system is tax-funded and free of charge. All data are mandatorily and automatically generated, updated, and linked via the unique personal identification number (CPR-number) given to all persons in Denmark at birth or upon immigration. After granted permission, data can be accessed using a secured platform guaranteeing anonymity. Data can be retrieved from 1977.

In 2014, we identified all persons in Denmark with MFS according to the Ghent-II criteria [2]. To do so, we retrieved CPR-numbers nationwide from all persons recorded in the Danish National Patient Registry with the diagnosis Q87.4 “Marfan Syndrome” or 759.80 “Arachnodactyly (Marfan syndrome)”, according to the International Classification of diseases 10th (ICD-10) and 8th (ICD-8), respectively, during 1977 to 2014. Registration of hospital and department was also retrieved. To verify the MFS diagnosis, we retrieved relevant medical records from these departments and scrutinized all records manually, as previously described [2]. In total, we originally identified 412 men and women with a registered MFS diagnosis. Of these, only 407 were alive in 1994 or later (1994 marks the beginning of the ICD-10 coding system). We have published data on morbidity and mortality in this population [15, 16].

Statistics Denmark provided an age and sex matched control population, matched 1:100. All controls were alive the day their relevant MFS person was diagnosed with Marfan syndrome. For controls and for MFS, we utilized the following information: Registrations of in- and outpatient diagnoses, surgery, and for cause of death, as well as the relevant dates. For this study we wanted to identify and describe the musculoskeletal morbidity of MFS, compared to a Danish control population. We focused on four different groups, namely rheumatic diseases, congenital deformities, dislocations, and musculoskeletal surgery. The end of the observation period was in 2017. We used the ICD-10 (from 1994 to 2017) for rheumatic diseases (M001-799), for congenital deformities (Q650-689), and for dislocations (relevant codes from S030-T039). For musculoskeletal surgery we used the Nordic Classification of Surgical Procedures (NOMESCO) that stared in 1996, primarily using KN codes (Musculoskeletal system). All four groups were subdivided into main groups and subgroups. For use of DMARD (Disease Modifying Antirheumatic Drug) we applied the Anatomical Therapeutic Chemical code (ATC) and the treatment procedure code as described previously [17]. The validity of some rheumatic diagnoses in the Danish registries has been investigated earlier, with positive predictive findings of approximately 88% for rheumatoid arthritis [18].

To describe the relation between musculoskeletal disease and the age at aortic dissection, we divided the patients with MFS in two groups, one where all had at least one registration of a musculoskeletal impairment from the Ghent II classification (scoliosis, pes planus, or pectus abnormalities), and one where none had such a musculoskeletal registration. Previously, we also identified all patients with MFS, who had an ICD-8 or 10 registration with an aortic dissection, corresponding to 80 persons [19].

### Statistics

Basic epidemiological data are given using medians and interquartile ranges (IQR).

For all patients, the time to the first registration in the Danish registers was analyzed using stratified Cox regression, where each patient with MFS and his or her matched control constituted one stratum. This regression model was used to investigate the association between the survival time of the patient and our selected variables. The outcome is presented as a Hazard Ratio (HR). A two-sided *p*-value of less than 0.05 was considered statistically significant.

For all analyses, time at risk started at the birth of the patient with MFS or at the start of the registry, whatever came first. Time at risk ended at the date of a relevant registration, which could be date of death, end of study (2017), or emigration (no longer registered as a citizen in Denmark), whichever came first. When comparing women with MFS with men with MFS, year of birth was used as a co-variate.

Age at the time of an aortic event (elective surgery or dissection) was log-transferred, and the t-test applied to compare those with and without a registration of a musculoskeletal diagnosis from the Ghent II classification.

Stata 16.1 for Windows (StataCorp LP, College Station, TX, USA) was used for all calculations.

### Ethics

The study was approved by the Scientific Ethical Committee (31,422) and the Danish Data Protection Agency (2011-41-6986). The European Union's General Data Protection Regulation does not accept any possibility of personal identification of cases; thus, Statistics Denmark prohibits specification of the exact number of cases with a given condition if less than four, and we therefore must report these as “< 4”.

## Results

Based on the 407 identified persons with MFS (194 women), we retrieved 40,700 controls (19,400 women) from the background population. At the end of 2017, 349 patients with MFS were alive and living in Denmark with a median age of 38 years (Table 1). The follow-up period covered 16,061 person years from birth to the end of the observation period.

**Table 1:** Epidemiological data from the Marfan syndrome cohort and the background population

	Number	Year of birth (IQR)	Age (years)* (IQR)	Number of years at risk	Number of deaths
Females with MFS	194	1976 (1961–1992)	39 (23–54)	7954	31
Males with MFS	213	1978 (1963–1994)	37.5 (21–50)	8107	27
Control, females	19,400	1976 (1961–1992)	40 (25–55)	–	1125
Control, males	21,300	1978 (1963–1994)	39 (22–52)	–	878

MFS, Marfan syndrome; IQR, Interquartile range

*Age at the end of the study, excluding deceased persons

– Not relevant

**Fig. 2:**
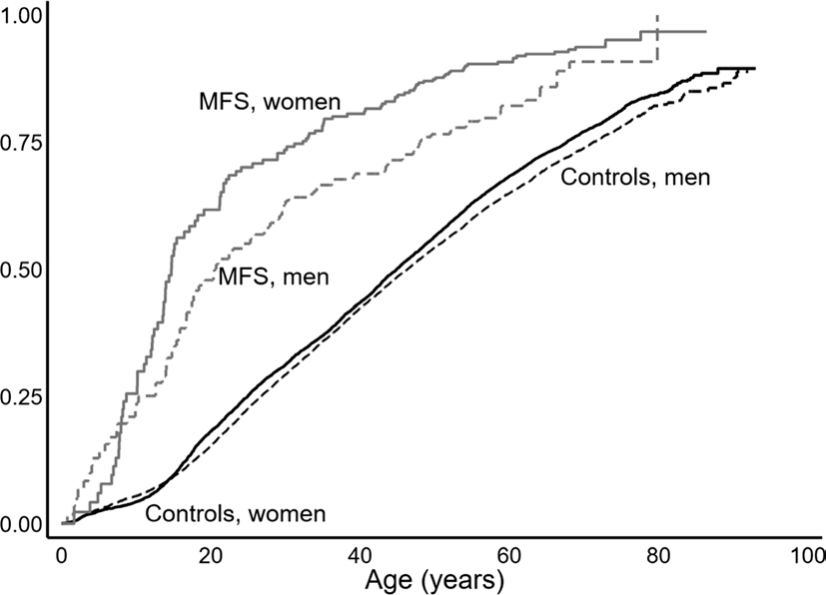
Musculoskeletal diagnoses in Marfan syndrome and the background population. Women with Marfan syndrome (solid gray line), men with Marfan syndrome (dashed gray line), control females (solid black line) and males (dashed black line) from the background population registered with of a musculoskeletal diagnosis, corresponding to ICD-10 code DM001 to DM799, for the first time

The burden of musculoskeletal disorders was quite significant, with an almost doubled risk (HR: 1.94 (1.69–2.24) of a registration of a musculoskeletal diagnosis (Table 2 and Fig. 2).

**Table 2:** Spinal and rheumatic disease in Marfan syndrome

Diagnoses	ICD-10 codes	Number of MFS versus controls	HR (95% CI)	HR (95% CI) MFS only, females versus males*	HR (95% CI), MFS with an aortic event versus MFS without
*Combined*^†^	M001–799	207/13,367	1.94 (1.69–2.24)	1.36 (1.03–1.80)	1.15 (0.79–1.69)
*Kyphosis, lordosis, and scoliosis*					
Kyphosis and lordosis	M400–405	6/25	23.8 (9.8–58.1)	0.52 (0.10–2.87)	N/A
Scoliosis, including	M410–419	68/216	36.7 (27.5–48.9)	2.72 (1.60–4.63)	1.91 (0.87–4.16)
Scoliosis in the young	M410–M411	27/102	31.2 (20.0–48.1)	4.30 (1.73–1.08)	0.62 (0.16–2.37)
*Malformation of the chest wall*					
Pectus excavatum	Q676	19/43	49.8 (28.7–86.4)	0.60 (0.24–1.53)	N/A
Pectus carinatum	Q677	21/16	140 (72.1–271.1)	0.77 (0.32–1.84)	1.52 (0.30–7.61)
*Congenital malformations of the lower extremities*	Q650–669	41/424	10.5 (7.6–14.5)	1.16 (0.62–2.16)	
Congenital deformity of the hip	Q650–659	4/169	2.54 (0.94–6.85)	3.83 (0.40–37.1)	N/A
Congenital deformity of the foot	Q660–669	37/258	15.6 (11.0–22.1)	1.02 (0.52–1.97)	0.55 (0.11–2.70)
Other valgus deformities of the foot	Q660–664	8/86	8.4 (3.9–18.2)	1.49 (0.33–6.73)	N/A
Congenital pes planus	Q665–666	17/72	26.0 (15.2–44.3)	1.59 (0.60–4.19)	N/A
*Inflammatory arthritis*					
Reactive arthritis	M02–03	N/A			N/A
Inflammatory polyarthritis	M05–14	12/752	1.71 (0.97–3.03)	0.51 (0.15–1.73)	1.42 (0.36–5.57)
Inflammatory spondylopathies	M45–46	N/A			0.35 (0.02–7.11)
*Degenerative and other musculoskeletal disorders*					
Osteoarthritis	M15–19	23/2052	1.12 (0.73–1.72)	1.50 (0.61–3.68)	0.74 (0.27–2.02)
Other joint disorders, including arthralgia	M20–25	78/4520	1.93 (1.55–2.42)	1.21 (0.77–1.90)	1.04 (0.57–1.92)
Spondylolisthesis	M431	4/136	3.34 (1.23–9.14)	3.83 (0.40–37.06)	0.93 (0.17–5.22)
Spondylosis and other spondylopathies	M47–49	7/645	1.16 (0.55–2.45)	6.39 (0.76–54.1)	0.61 (0.06–6.27)
Intervertebral disc disorders	M50–51	7/1559	0.47 (0.22–0.98)	1.32 (0.29–5.99)	1.92 (0.93–3.99)
Dorsalgia	M53–54	37/2.301	1.76 (1.26–2.43)	1.44 (0.75–2.76)	0.74 (0.27–2.02)
*Soft tissue disorders*					
Disorders of synovium and tendons	M650–689	18/1686	1.12 (0.71–1.79)	2.20 (0.82–5.88)	0.81 (0.35–1.86)
Other soft tissue disorders	M700–790	38/3504	1.14 (0.83–1.57)	1.50 (0.78–2.89)	N/A

Diseases of the spine and rheumatic disease diagnoses from the International Classification of Diseases, 10th edition (ICD-10), combined and in main groups (in bold) and in subgroups in patients diagnosed with Marfan syndrome (MFS) and in their controls as well as in females with MFS compared to males with MFS. One patient may have several diagnoses

*Year of birth was used as co-variate. HR: Hazard ratio. N/A not available due to limited number of events

^†^Each person could have a registration in more than one main group or subgroup

**Fig. 3:**
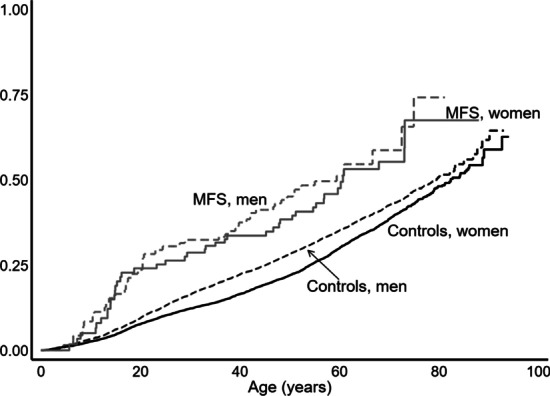
Musculoskeletal surgery in Marfan syndrome. Proportion of women with Marfan syndrome (MFS) (solid gray line), men with MFS (dashed gray line), control females (solid black line) and males (dashed black line) from the background population registered with musculoskeletal surgery, corresponding to NOMESCO code KN, for the first time

Moreover, the proportion of patients with MFS that underwent musculoskeletal surgery was also significantly increased (HR: 1.76 (1.43–2.16)) (Table 3 and Fig. 3).

**Table 3:** Registrations of musculoskeletal surgery in Marfan syndrome

Surgery	NOMESCO	Number of MFS/controls	HR MFS versus controls (95% CI)	HR MFS only, women versus men * (95% CI)
Combined^†^	KN	94/5949	1.76 (1.43–2.16)	0.90 (0.60–1.35)
Non-spinal surgery		83/5786	1.57 (1.27–1.95)	0.85 (0.55–1.31)
*Surgery of the spine*				
Spine and neck	KNA	17/248	7.5 (4.6–12.3)	2.06 (0.76–5.58)
Scoliosis	KNAG71-75	14/87	17.6 (10.0–31.1)	2.71 (0.85–8.67)
	KNAT21-25			
*Surgery of the chest wall*				
Correction pectus carinatum	KGAF00	6/ < 4	> 200	N/A
Correction pectus excavatum	KGAF03	7/9	77.5 (28.9–208.1)	N/A
*Upper extremity surgery*				
Shoulder and upper arm	KNB	8/788	1.07 (0.53–2.15)	0.87 (0.21–3.60)
Elbow and forearm	KNC	14/862	1.68 (0.99–2.85)	0.28 (0.08–1.05)
Wrist and hand	KND	12/1314	0.95 (0.54–1.68)	1.01 (0.32–3.18)
*Pelvic and lower extremity surgery*				
Pelvis	KNE	< 4/63	N/A	N/A
Hip joint and thigh	KNF	17/617	2.95 (1.82–4.78)	1.11 (0.42–2.88)
Hip arthroplasty	KNFB	4/222	1.89 (0.70–5.10)	1.05 (0.15–7.51)
Knee and lower leg	KNG	25/2289	1.13 (0.76–1.68)	0.69 (0.31–1.54)
Ankle and foot	KNH	28/996	3.08 (2.12–4.49)	1.40 (0.66–2.96)
Resection of joint, arthroplasty and arthrodesis in ankle and foot	KNHG	14/88	16.9 (9.6–29.8)	0.97 (0.34–2.789)
Osteotomy of ankle or foot	KNHK50-69	7/151	4.80 (2.25–10.24)	1.48 (0.33–6.62)
Myotomy or tenotomy of ankle or foot	KNHL39	6/41	17.3 (7.3–41.4)	2.17 (0.39–12.0)
Correction of deformity of ankle or foot using external or internal fixation	KNHT49	< 4/13	17.7 (3.9–79.7)	N/A
Removal of internal fixation device from ankle or foot	KNHU49	14/178	8.3 (4.8–14.4)	1.41 (0.49–4.08)

Registrations of surgery according to the Nordic Classification of Surgical Procedures (NOMESCO) in patients diagnosed with Marfan syndrome and in their controls, combined and divided into eight main groups (in bold), and into subgroups. Further, data on the correction of pectus carinatum and pectus excavatum are given. One patient may have several registrations of surgery

*Using year of birth as a co-variate. HR, Hazard ratio. N/A not available due to limited number of events ^†^Each person could have a registration in more than one main group or subgroup

### Scoliosis, spondylolisthesis, and spinal surgery

More than one out of six (69/407) patients with MFS were registered with an ICD-10 diagnosis of scoliosis, corresponding to a HR of 37 (Table 2). Significantly more women with MFS were affected compared to men (Table 2). Furthermore, 3% (14/407) with MFS were registered with having corrective spinal surgery due to scoliosis and additionally 17 patients with MFS were registered with corrective neck or spinal surgery (Table 3). The median age at the time of scoliosis surgery was 10 years for MFS and 20 years for the controls. The median number of registrations of scoliosis surgery was 2 (IQR 1–4) in MFS and 1 (IQR 1–1) in controls, (Kruskal Wallis test: *p* < 0.01). No patients with MFS were registered with complications such as infections in tendons, joints, discs, and bones, corresponding to the code NOMESCO “KNAS”, whereas six of the controls had such a registration. Dislocations of the spine were limited in MFS compared to controls (Table 4). Spondylolisthesis was only registered in approximately 1 percent of the patients with MFS (Table 2).

**Table 4:** Dislocations of the joints in Marfan syndrome

Diagnoses	ICD-10 codes	Number of MFS versus controls	HR (95% CI)	HR (95% CI) MFS only, women versus men*
Combined	All below	149/13617	1.17 (1.00–1.38)	0.69 (0.50–0.96)
Jaw dislocation	S030–S035	11/1574	0.72 (0.40–1.29)	0.98 (0.30–3.23)
Cervical dislocation	S130–S136	7/1307	0.54 (0.26–1.15)	0.85 (0.19–3.80)
Shoulder dislocation	S430–S437	11/968	1.22 (0.68–2.22)	0.51 (014–1.75)
Elbow dislocation	S530–S534	7/638	1.12 (0.53–2.36)	0.20 (0.02–1.70)
Finger dislocation	S630–S637	41/3999	1.07 (0.79–1.46)	0.54 (0.28–1.04)
Hip dislocation	S730–S731	< 4/96	N/A	N/A
Dislocations, knee/patella	S830–S837	40/3724	1.13 (0.83–1.55)	0.75 (0.40–1.40)
Dislocations, ankle/foot	S930–S936	77/7130	1.13 (0.90–1.42)	0.96 (0.61–1.50)
More than one dislocation	T030–T039	< 4/17	N/A	N/A

Diagnoses related to dislocations of the joints from the International Classification of Diseases, 10th edition (ICD-10), combined and in main groups in patients with Marfan syndrome (MFS) and in their controls, as well as in women with MFS compared to men with MFS. One patient may have several diagnoses

^*^Using year of birth as a co-variate. HR, Hazard ratio. N/A not available due to limited number of events

Each person could have a registration in more than one main group or subgroup

### Chest wall deformities

Deformity of the chest wall was the most common congenital skeletal deformity. One out of 11 (37/407) with MFS were registered with either pectus excavatum (HR: 49.8 (28.7–59.3)) or pectus carinatum (HR: 140 (72.1–271.1)) (Table 2) and 13/37 had pectus surgery (Table 3). None were registered with aortic surgery after pectus surgery. The median age at pectus surgery was 17 years for MFS and 16 years for the controls. There was no difference between men and women with MFS.

### Disease in lower extremities and feet

Registration of congenital malformations of the hip was not significantly more common in MFS (HR: 2.54 (0.94–6.85). Even though, the diagnosis of protrusion acetabuli was almost not used in the register, significantly more patients with Marfan syndrome indeed had surgery of the hip (HR: 2.95 (1.82–4.78)) (Table 3).

More than one out of six with MFS (66/407) had a registration with a deformity of the foot. This being either pes planus (HR: 26.0 (15.2–44.3), congenital deformity of the foot HR: 15.6 (11.0–22.1), or congenital valgus deformities in foot HR: 8.4 (3.9–18.2), whereas this only applied to 585 individuals in the control group (1.4%) (Table 2).

### Rheumatic disease

At the age of 25 years, 63% of persons with MFS and 24% of the controls had at least one registration with a rheumatic diagnosis. The risk of being registered with any rheumatic disorder was almost doubled (HR: 1.9 (1.7–2.2)) in MFS compared to controls, with a significantly increased risk in women compared to men (Table 2). Most rheumatic diagnoses were related to low back pain or joint pain (Table 2). Inflammatory rheumatic disorders were not more common in patients with MFS, and none was treated with a DMARD.

### Non-spinal orthopedic surgery

The combined risk of being registered with non-spinal orthopedic surgery (here defined as NOMESCO code KN) was significantly increased in MFS compared to controls (HR: 1.57 (1.27–1.95)) (Table 3), and this applied to three out of eight main groups. At the age of 25 years, 28% of persons with MFS and 12% of the controls had at least one registration with orthopedic surgery. Women and men with MFS were equally affected (Fig. 3). The major surgical areas were hip surgery and surgery of the ankle and foot (Table 3).

### Dislocations

The registration of any dislocation was seen with a borderline increased HR of 1.17 (1.00–1.39, *p* = 0.055) in MFS. Congenital dislocations of the hip were quite rare (Table 4). In MFS, the risk of being registered with a dislocation was significantly lower in women compared to men (Table 4).

### Cancer

None in the MFS cohort were registered with cancer of the bone or connective tissue.

### Medication

None in the MFS cohort were registered with the use of DMARDS, or in-hospital or out-patient clinic glucocorticoid injections.

### Surgery according to phenotype

Presence of at least one of the major musculoskeletal phenotypes (scoliosis, pectus deformities, and pes planus) was registered as a diagnosis in 102/407 (25.1%) of patients with MFS and in 544/40700 (1.4%) of controls. Interestingly, ten patients underwent corrective surgery without being recognized and diagnosed with MFS until later in life. However, none one of these patients were registered with phenotypical characteristics associated to MFS.

### Aortic dissection and musculoskeletal phenotype

Eighty patients with MFS had an aortic dissection, and 102 had at least one musculoskeletal registration from the Ghent II classification. Among the patients with MFS and an aortic dissection, those with at least one musculoskeletal Ghent II registration, had an age at dissection of 34.3 years, whereas age at dissection in those without a musculoskeletal Ghent II registration was 45.1 years (*p* < 0.01). Detailed information on phenotype and aortic events is presented in Table 2.

## Discussion

The main finding of the present study was an extensive burden of musculoskeletal diseases in patients with Marfan syndrome, primarily related to the spine and chest wall. At the age of sixty years, more than half of the population with MFS had been diagnosed with a musculoskeletal disorder and many needed surgical treatment. In addition, it seemed that patients with a more abnormal musculoskeletal phenotype developed aortic dissection at an earlier age than patients with a phenotype without obvious musculoskeletal disease.

A national registry-based study uses automatically collected data that reflect clinical practice and registered events related to an entire population. This means that the presented data reflect outpatient visits, admissions, or surgical procedures and not issues assessed in a research setting. So, if the patient does not choose to see a physician due to a musculoskeletal problem she or he will not be registered in the present dataset. Moreover, minor abnormalities in the spine or chest wall may not have been registered if they were considered mild or were without symptoms.

In general terms, the musculoskeletal problems in MFS are caused by a ligamentous laxity and excessive longitudinal growth of the tubular bones [4, 6, 20]. This has effect on the weightbearing joints and on the spine where scoliosis is a common feature in the MFS phenotype [21].

Compared to the background population, a relatively high number of patients with MFS had spinal surgery and it was performed at significantly younger age and mostly in women. These findings are in accordance with other observational studies [13, 21]. Scoliosis bracing for children and adolescents can be effective but growth-friendly spinal surgery may be necessary in many cases which includes staged surgery and growth friendly spine implants [22, 23]. Both the increased risk of implant failure in these cases and the need for multiple surgical stages is reflected in our findings where the number of surgical procedures was twice as high as in the background population [21, 22]. However, we did not see a significantly higher number of surgical complications among patients with MFS, as previously reported [23]. Spine surgery in patients with MFS has changed over the years with a decline in the number of procedures and a trend towards a posterior surgical approach [13, 23]. In Denmark, the caretaking of patients with MFS is centralized to two hospitals, which ensures the highest experience among surgeons engaging in this rare condition. Specialized rehabilitation of these patients must also be a key issue to ensure the best possible result [24].

Westphal et al. reported a pectus carinatum prevalence of 0.7% and a prevalence of pectus excavatum 1.3% in a cohort of 1332 normal children aged 11–14 years [25]. Among patients with MFS, we found a much higher number of patients with chest wall deformities (around 10 percent) and one in four cases chose to have corrective surgery. Surgical correction of chest wall deformities with the NUSS-procedure began in 2001 in Denmark and during the first 15 years over 1700 patients underwent this minimal invasive procedure [26]. With the NUSS-procedure, a metal bar reaching from one midaxillary line to the other is inserted beneath the sternum with a minimal invasive surgical technique [27]. Considering the low incidence of chest deformities reported in the literature and an annual number of births in Denmark of around 50,000, the number of NUSS-procedures is relatively high and could indicate that a large pool of patients had been offered surgery during the first years after introducing the NUSS-procedure in Denmark [25, 26, 28]. Naturally, this new interest will also have included patients with MFS, which can have increased the incidence of NUSS-procedures during the follow-up period of the present study [12]. After the NUSS-procedure, the metal bar has to remain in this position for approximately three years which hinders quick access to the aorta in case of an aortic dissection [12]. It is therefore important to ensure the correct timing of corrective chest surgery in patients with MFS or to combine open heart and chest surgery in one procedure [29]. We did not find any cases with a need for aortic surgery after a NUSS-procedure, which indicates that these patients were young and well selected.

Protrusio acetabuli is an intrapelvic displacement of the acetabulum and femoral head and is found in more than 75% of patients with MFS [30, 31]. Even though it is a feature in the Ghent II nosology it is not necessary to diagnose if the phenotype is otherwise obvious [3]. This is probably the reason why the prevalence was very low in this cohort. Protrusio acetabuli can lead to hip dysfunction and impingement that may need surgical treatment [31, 32]. Over time, protrusio acetabuli can also cause osteoarthritis and a need for hip replacement [31, 32]. Accordingly, there was a significantly increased number of hip surgeries in our population. However, the number of hip arthroplasties was not higher than in the background population. This clearly shows that hip problems are quite common in MFS. With an increasing life span of the patient with MFS there will be more patients with a need for hip replacement in their senior years [15, 31].

Flatfoot and hindfoot deformities are also common in MFS [5]. The growth of the metatarsals and phalanges and the ligament laxity in the foot causes loss of the medial arch [33]. The primary treatment is non-surgical with shoe orthoses and physical therapy exercises [34]. However, surgery may be necessary to relieve pain [10]. There is not much literature regarding surgical techniques and no consensus on how to approach this problem with surgery [34, 35]. This contrasts with our findings, where we saw a very high number of surgical foot and ankle treatments including arthroplasty and arthrodesis. This surgical practice in Denmark most likely reflect the magnitude of feet problems in MFS but is not supported by a recommendation that surgery should be preferred in patients with MFS [34].

Ligament laxity should lead to joint dislocations. However, this was not the case in this cohort. Patients with MFS did not have significantly more joint dislocations in the fingers, shoulders or in the lower extremities (i.e., patella luxations). Patients with MFS may have a more sedentary lifestyle where they refrain from sports, either due to pain and disabilities or concerns about aortic dissection [10, 36]. This will obviously minimize the number of traumatic dislocations and is probably why the number of dislocations was low.

Some inherited disorders may be mistaken as a rheumatic disease and the starting point of treatment will therefore be at a rheumatologist [37]. In the present study, patients with MFS were mainly seen by a rheumatologist due to musculoskeletal pain, which is a major issue in MFS [10, 38]. From a previous study in this population, we have shown that more than one in four patients use analgesics (including opioids) on a regular basis [38]. We did not find an increased prevalence of inflammatory rheumatic disorders compared to the background population.

The finding of ten patients with MFS who underwent corrective surgery due to a musculoskeletal disorder without being diagnosed is quite remarkable. This result should be a reminder to all surgeons and rheumatologist in pediatric or adult care, to be aware of patients with abnormal phenotypes that could have MFS [5, 9, 39]. For these patients it could be lifesaving to get the diagnosis right, especially since patients with a more profound musculoskeletal phenotype experienced aortic dissection ten years earlier than patients without obvious phenotypical features [19, 40].

### Limitations

A register study has several limitations and only provides data on what is found and registered by doctors. A nationwide set-up stretched over many years is never as accurate as a single center cohort where patients are being taken care of by specialists. We also do not have the patients’ genotypes. We cannot exclude that some patients in Denmark are not yet diagnosed or have already died without being diagnosed correctly. It is also likely that we primarily included case patients with an obvious phenotype and there may be an under-registration of some types of trivial issues like minor abnormalities of the chest and spinal column.

## Conclusion

The extent of musculoskeletal disease is quite significant in Marfan syndrome, and many will need corrective surgery during their life span. Surgeons should be aware of undiagnosed patient with Marfan syndrome when treating patients with a Marfan syndrome like phenotype.

## Data Availability

The datasets analyzed during the current study are available from the corresponding author on reasonable request.
